# Prefrontal-hippocampal theta coherence, sharp wave ripples, and bursts of cortical unit activity underlie choices and encoding in the radial arm maze

**DOI:** 10.1186/1471-2202-16-S1-P139

**Published:** 2015-12-18

**Authors:** Maxym V Myroshnychenko, Christopher C Lapish

**Affiliations:** 1 Program in Neuroscience, Indiana University, Bloomington, IN 47401, USA; 2 Addiction Neuroscience Program, Indiana University-Purdue University, Indianapolis, IN 46202, USA

## 

**Figure 1:**
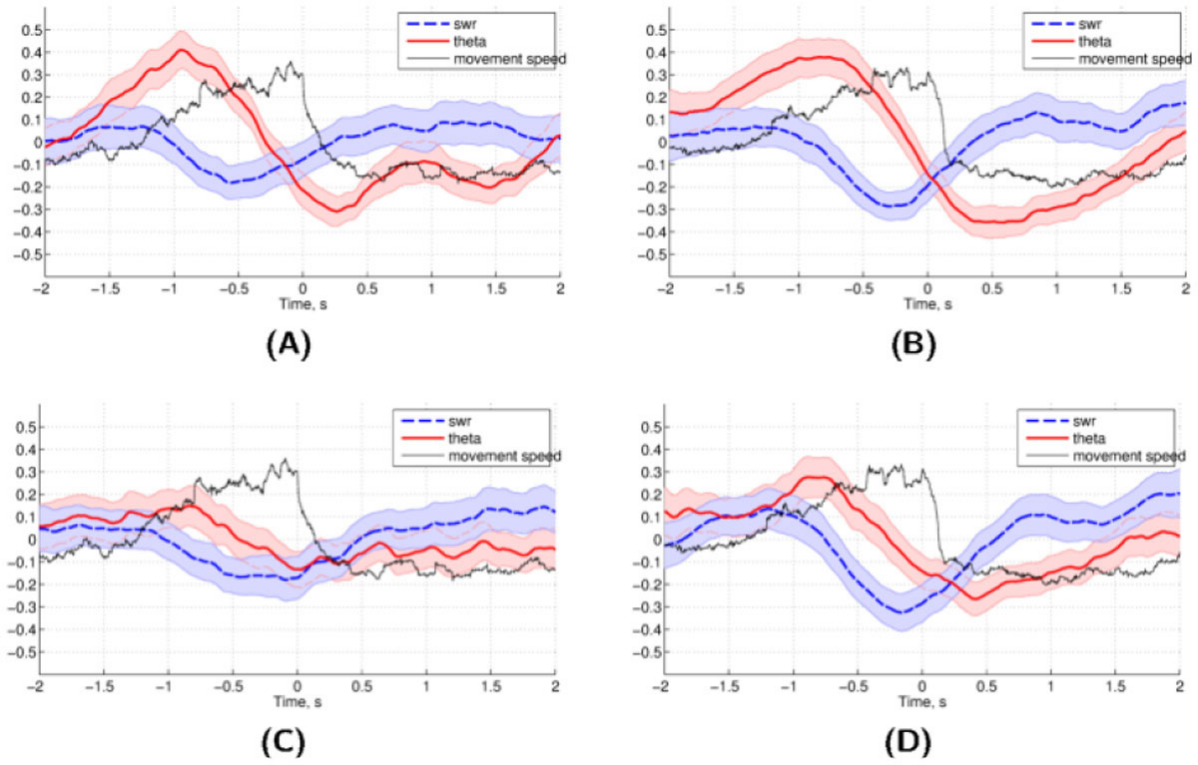
**Average hippocampal and prefrontal theta and SWR power centered on reward acquisition**. During test choices (B, D), PFC (D) activity resembles that in HC (B). On the other hand, during training (A, C), HC (A) shows the pattern of activity associated with reward sites, and PFC (C) does not. Shaded regions, 95% confidence intervals

The radial arm maze (RAM) is a foraging task that is often used to assess executive function guided decision-making [1-3]. Optimal foraging strategies on this task require the integration of executive and memory systems, which include retrospective and prospective codes. To explore the neural basis of decision-making during RAM performance, the current study acquired multielectrode single-unit and local field potential (LFP) recordings simultaneously in hippocampus (HC) and anterior cingulate (ACC). Initially, rats were presented with four of the eight total maze arms open at once ("training phase"), and the rest of the arms were opened up after the subjects collected the rewards and a one-minute delay was completed ("test phase"). Any arm re-entries were counted as errors, and only trials with one test phase error or less were used in the analysis. ACC unit firing was elevated at choice points and reduced at reward points. Moreover, both ACC and the HC showed elevated theta power shortly prior to the reward point and sharp wave ripples shortly after reward acquisition on correct choices only, which is consistent with findings in other choice tasks [4,5]. These observations held during test phase (Figure 1, right panels) and were disrupted during the training phase in ACC, but not the HC (Figure 1, left panels). It has been suggested that HC sharp wave ripples contain episodes of replay of visited locations and theta of locations ahead of the animal [4,5] - information necessary for decision-making using both prospective and retrospective codes. Theta and ripples are present in the HC during both training and test phases, consistent with lesion evidence that hippocampus is necessary for both prospective and retrospective strategies [1]. In ACC, theta and ripples are only present during test phase and not during training phase, which is in line the evidence showing that prefrontal cortex is only necessary for prospective coding [1]. These task phase-dependent observations may help explain how HC and ACC networks integrate information related to prospective and retrospective codes.
